# Robust CRISPR/Mb2Cas12a genome editing tools in cotton plants

**DOI:** 10.1002/imt2.209

**Published:** 2024-06-04

**Authors:** Fengjiao Hui, Xu Tang, Bo Li, Muna Alariqi, Zhongping Xu, Qingying Meng, Yongxue Hu, Guanying Wang, Yong Zhang, Xianlong Zhang, Shuangxia Jin

**Affiliations:** ^1^ Hubei Hongshan Laboratory, National Key Laboratory of Crop Genetic Improvement Huazhong Agricultural University Wuhan China; ^2^ Chongqing Key Laboratory of Plant Resource Conservation and Germplasm Innovation, Integrative Science Center of Germplasm Creation in Western China (Chongqing) Science City, School of Life Sciences Southwest University Chongqing China; ^3^ Institute of Nuclear and Biological Technology, Xinjiang Academy of Agricultural Sciences Xinjiang Key Laboratory of Crop Biotechnology Urumqi China

## Abstract

The efficiency and accuracy of the CRISPR/Mb2Cas12a system were demonstrated in cotton, achieving an efficiency of over 90% at target sites. Notably, Mb2Cas12a exhibited significant tolerance under different temperatures ranging from 22°C to 32°C. Additionally, the Mb2Cas12a system revealed effective editing at more relaxed VTTV PAM sites in the cotton genome, which expanded the genome editing range by approximately 2.6‐fold than the wide‐type LbCas12a. Finally, a multiplex genome editing system was also developed based on Mb2Cas12a, enabling simultaneous editing of eight target sites using a single crRNA cassette.

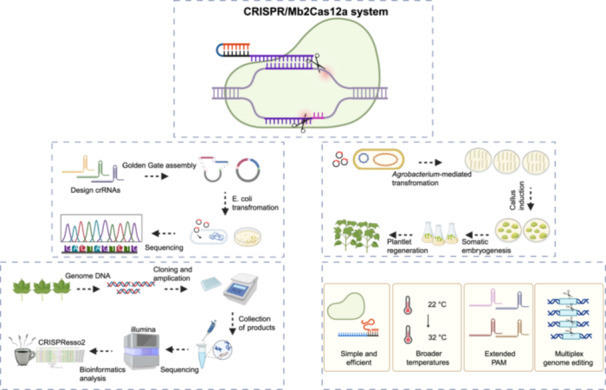

CRISPR/Cas9 system has sparked a biotechnological revolution in various fields, serving as a milestone for biotechnology by enabling target mutagenesis with unparalleled simplicity, high efficiency, and precision [1, 2, 3, 4]. The CRISPR/Cas12 system possesses certain features that make it a promising tool for genome editing [5]. Recently, CRISPR/Mb2Cas12a was described to perform effective editing in plants. Importantly, Mb2Cas12a can recognize not only TTTV (V = A, C, G) PAMs but also identify the more relaxed VTTV PAM to expand the editing range of the whole genome [6]. However, this system has not been tested in cotton plants yet.

Upland Cotton (*Gossypium hirsutum*) is an allotetraploid with a large and complex genome (AADD, 2n = 4x = 52) of approximately 2.5 Gb, where most genes are duplicated. Although several CRISPR/Cas12a systems have been developed for various plant species, only a limited number of Cas12 proteins have been utilized in cotton genome editing. Most CRISPR/Cas studies used a mixed dual promoter system in cotton [7, 8]. Whether other types of Cas proteins and single RNA (sgRNA)/crRNA expression systems can enhance editing efficiency in cotton remains to be investigated. Furthermore, multiplex genome editing systems have been developed using Cas9 and Cas12a in plants, but the effectiveness of achieving highly efficient editing at multiple sites in cotton still needs to be tested [9, 10]. In light of this, the present study aims to establish the CRISPR/Mb2Cas12a system in cotton, evaluate different crRNA expression cassettes, and finally assess the feasibility of applying multiplex genome editing.

## RESULTS AND DISCUSSION

### CRISPR/Mb2Cas12a enables highly effective targeted mutations in cotton

**Figure 1:**
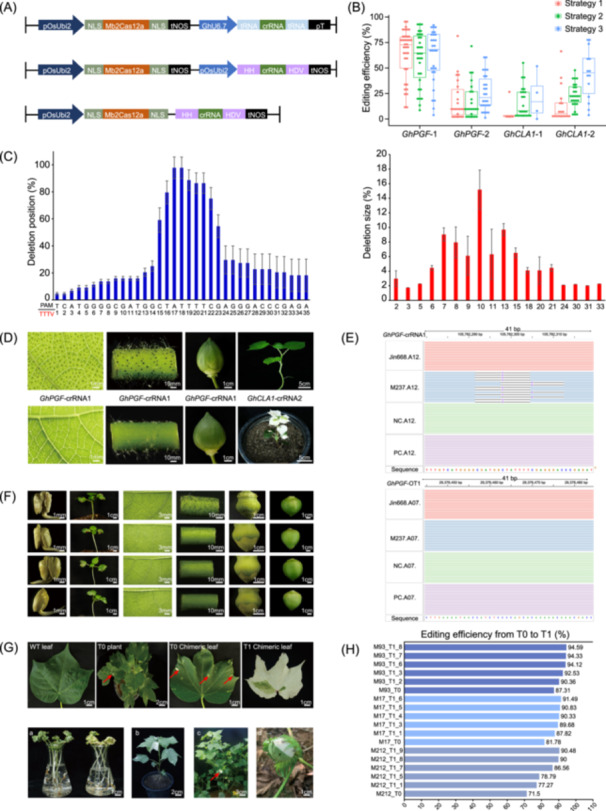
Development of high efficiency CRISPR/Mb2Cas12a system in cotton. (A) A schematic illustration of the T‐DNA region of the CRISPR/Mb2Cas12a binary vector for single site targeted editing. These elements include p*OsUbi2*: rice ubiquitin promoter, NLS: nuclear localization signal, pT: polyT, *GhU6.7*: cotton U6.7 promoter, HH: hammerhead ribozyme, HDV: hepatitis delta virus ribozyme, and tNOS: nopaline synthase terminator. (B) Editing efficiency (%) at target sites of *GhPGF* and *GhCLA1* with different vectors. All data were obtained from next‐generation sequencing (NGS). Each point represents the editing efficiency of an independent sample. Each dot represents a biological replicate. (C) Frequency of DNA deletion position and size at the target site of *GhPGF*‐crRNA1 induced by Mb2Cas12a. The PAM sequence is highlighted in red, and crRNA is depicted in black. Error bars represent a biological replicate. (D) Phenotype of the T0 cotton plants with the target mutations in *GhPGF* and *GhCLA1*. The black dots represent the gossypol glands on cotton plants. The T0 knockout plants targeting *GhCLA1*‐crRNA2 exhibited a chimeric phenotype with scattered white areas on green leaves. The figures in the first row represent the wild type (WT). (E) Evaluation of potential off‐target editing through whole‐genome sequencing technology. Sequence alignments of *GhPGF*‐crRNA1 target site and off‐target site *GhPGF*‐OT1 visualized through Integrative Genomics Viewer (IGV). (F) Phenotypic characterization of T1 lines at the *GhPGF*‐crRNA1 site. Black dots indicate the presence of gossypol glands on the surface of T1 seed, seedling, leaf, petiole, flower buds, cotton bolls, and WT control samples. (G) T1 cotton plants with mutations in *GhCLA1*‐crRNA2 site showed leaf bleaching in different environments: (a) indoors, (b) greenhouse, (c) field, and (d) cotton peach grown in the field. (H) Determination of the target mutation efficiency at the *GhPGF*‐crRNA1 target site in T1 lines using NGS.

First, we constructed an Mb2Cas12a expression vector termed Strategy 1. In Strategy 1, Mb2Cas12a and crRNA cassette containing 21 nt direct repeat fused with 23 nt guide RNA were driven by a Pol III promoter (*pOsUbi2*) and Pol II promoter (*pGhU6.7*), respectively. The crRNA was spaced with tRNA (Figure 1A) [11]. To assess the efficiency of the Mb2Cas12a system, we selected the visual reporter genes *GhPGF* and *GhCLA1* as the target genes. Transgenic cotton plants were generated through *Agrobacterium*‐mediated transformation (Figure S1). Subsequent next‐generation sequencing analysis revealed a high editing efficiency of up to 94.62% at the *GhPGF*‐crRNA1 target site in T0 plants (Figures 1B and  S3 and Table S1). The main editing window of Mb2Cas12a ranged from 13 to 26 nt downstream of the PAM sites. Only DNA deletions were detected at all target sites of edited T0 plants, with deletion sizes mainly ranging from 7 to 18 bp (Figure 1C). These findings were consistent with our previous verification of LbCas12a in cotton, indicating a similar editing pattern within the Cas12a family [7]. We observed a significant decrease in the density of gossypol glands in both the leaf and stem tissues, leading to a considerable number of plants lacking gossypol glands (Figure 1D). Meanwhile, plants carrying the *GhCLA1*‐crRNA2 component exhibited a partially bleached phenotype on their leaves, with editing efficiency reaching up to 71.34% (Figure 1B,D). Notably, all the tested T0 plants exhibited a chimeric phenotype because the homozygous mutants of *GhCLA1* were unable to survive in the soil. Furthermore, the efficient editing was also detected at other two target sites, *GhPGF*‐crRNA2 and *GhCLA1*‐crRNA1 (Figures 1B and  S4−S7).

Next, two other expression systems were also evaluated in cotton, Strategy 2 and Strategy 3. In Strategy 2, the Mb2Cas12a and crRNA arrays were driven by the *pOsUbi2* individually. The crRNA array was processed by Hammerhead ribozyme (HH) and Hepatitis Delta Virus (HDV) ribozyme. In Strategy 3, the Mb2Cas12a and crRNA arrays were driven simultaneously by a single *pOsUbi2*, named single transcript unit (STU) system (Figure 1A) [12]. We selected the same four targeted DNA sequences used in Strategy 1 to generate transgenic plants. Like Strategy 1, both Strategy 2 and Strategy 3 achieved high editing efficiency up to 94.33% and 91.95% at the *GhPGF*‐crRNA1 site (Figure 1B and Table S1), respectively, showing similar editing patterns and obvious phenotypes as those of Strategy 1 (Figure S1). Notably, significant editing events were also detected at the *GhCLA1*‐crRNA2 target site, with editing efficiencies of 47.81% and 78.18%, respectively (Figure 1B). Efficient editing was achieved at two other target sites as well (Figures S3−S6).

Our study demonstrated that CRISPR/Mb2Cas12a is a highly accurate and efficient genome editing tool in cotton plants. It can be successfully utilized in combination with three different crRNA arrays: tRNA‐crRNA‐tRNA, HH‐crRNA‐HDV, and STU. Additionally, our findings indicated that the editing efficiency remained essentially consistent regardless of whether a Pol II or Pol III promoter was used in the expression cassette containing a single crRNA.

### Mb2Cas12a demonstrates targeting specificity and genetic stability in cotton

To evaluate the specificity of Mb2Cas12a, Sanger sequencing and whole genome sequencing analyses demonstrated that no off‐target mutation was detected at any predicted off‐target sites (Figures 1E and  S2). Furthermore, the phenotypes and genotypes at the edited loci in cotton plants were faithfully inherited from the T0 to the T1 progeny, and some new editing events were observed in the T1 lines due to the presence of Mb2Cas12a protein in some T1 plants, which generated some new editing besides the existing editing loci (Figures 1F−H and S2). Remarkably, two transgene‐free T1 plants (pgf‐1 and pgf‐2) were successfully obtained due to genetic segregation with targeted mutations exhibiting gossypol glandless plants, which is one of the major advantages of genome editing technology over the traditional transgenic strategy (the predicted phenotypes/traits relay on the transgene integration in the genome of the host cells) (Figure S2).

### Mb2Cas12a exhibits wide temperature adaptability in cotton genome editing

**Figure 2:**
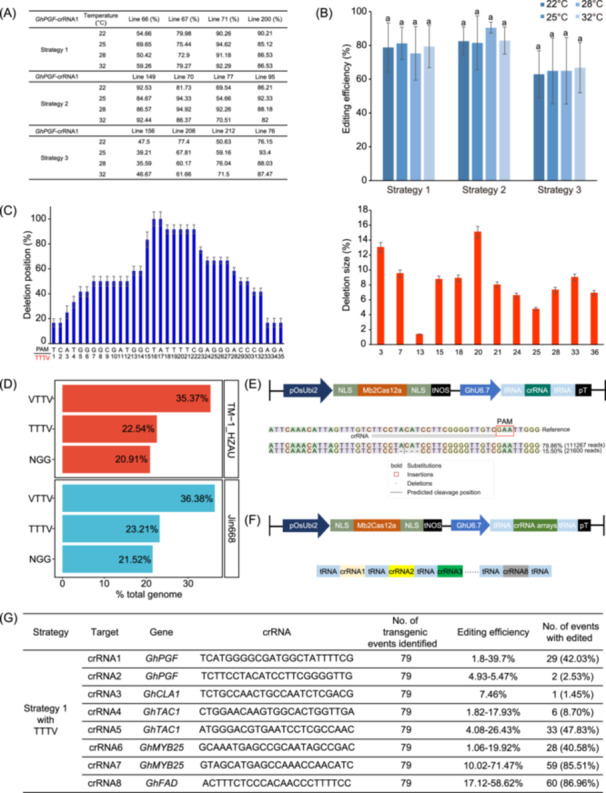
Wide temperature tolerance and multiplex genome editing by Mb2Cas12a in cotton. (A) Selection of 12 edited T0 plants with varying editing efficiencies from three strategies for temperature treatment under 22–32°C. Four edited plants were tested under each strategy. (B) Statistical analysis of edited plants under different temperatures. Next‐generation sequencing (NGS) data demonstrated that there was no significant difference in the editing efficiency of plants subjected to different temperature treatments. Each dot represents at least three biological replicates. Data are presented as mean values ± SD. Statistical differences were analyzed using Student's *t*‐test; The letter “a” represents difference with no significance, *p* > 0.05. (C) Analysis of mutation profiles specifically under 22°C using Strategy 1. (D) Genome‐wide analysis of targetable PAM sites recognized by Mb2Cas12a in cotton, including TM‐1_HZAU and Jin668 genomes. The numbers represent the proportion of PAM sequences recognized by Mb2Cas12a in the whole genome. N = A, T, C, and G. V = A, C, and G. (E) Effective editing capability of Mb2Cas12a at TTV PAM site. Schematic illustration of the T‐DNA region of the CRISPR/Mb2Cas12a binary vector containing TTV PAM. The image showed the result of NGS. The PAM sequence is highlighted in the red box. (F) Schematic illustration of the multiplex Mb2Cas12a editing system. The expression cassette comprises an Mb2Cas12 protein and eight crRNAs targeting five different genes. Key elements include *pOsUbi2*: rice ubiquitin promoter, NLS: nuclear localization signal, *pGhU6.7*: cotton U6.7 promoter, and pT: poly T sequence. (G) Summary of editing efficiencies of Mb2Cas12a at eight TTTV PAM sites in transgenic calli.

To investigate the editing activity of Mb2Cas12a under different temperature conditions, we selected a total of 12 T0 edited plants targeting the *GhPGF*‐crRNA1 for the temperature adaptability test. These plants were subjected to temperature treatments at 22°C, 25°C, 28°C, and 32°C for 7 days (Figure 2A). Interestingly, we found that Mb2Cas12a was slightly affected by lower temperatures, and considerable editing activity was detected under 22°C condition. For example, the editing efficiency of M71 (a plant generated from Strategy 1) was 90.26%, 94.62%, 91.18%, and 92.29% at 22°C, 25°C, 28°C, and 32°C, respectively, indicating no significant difference under four temperatures in cotton (Figure 2B). Analysis of the mutation profiles in these 12 edited plants revealed that the deletion position and sizes of the Mb2Cas12 were not affected by varied temperatures (Figures 2C and S8). We also carried out the same temperature treatments on T1 lines targeting the other three sites and no significant differences were observed (Figure S9).

Previous reports have indicated that nucleases from the Cas12 family are sensitive to temperature [13, 14]. Differences in editing efficiency have been observed among different plant species, which may be primarily attributed to the lower temperature (25–30°C) required for most plant tissue culture processes reducing the activity of Cas12 nuclease. Nevertheless, our research findings indicate that Mb2Cas12a displays excellent adaptability to varying temperatures and shows significant editing efficiency even under lower temperature conditions. Therefore, Mb2Cas12a holds great promise as a friendly genome editing technology for a vast number of plant species that prefer cooler growing conditions.

### Mb2Cas12a greatly expands the scope of cotton genome editing

To assess the targeting scope of Mb2Cas12a, we conducted a comprehensive whole genome‐wide PAM analysis in cotton. Results showed that the NGG PAM sites recognized by SpCas9 cover only 21.52% of the Jin668 (the most widely used genotype for cotton genetic transformation) reference genome. In comparison, the TTTV PAM sites recognized by the LbCas12a cover 23.21% of the genome, whereas Mb2Cas12a with its TTV PAM sites can target up to 59.59% of the cotton genome, which represents a 2.8‐fold increase compared to SpCas9 and a 2.6‐fold increase compared to LbCas12a (Figure 2D). Additionally, we employed Strategy 1 to construct the expression vector targeting *GhPGF*, and we achieved an editing efficiency of ~15.50% with the ATTV PAM site, resulting in 4‐bp deletion (Figure 2E). Notably, these ATTV PAM sites were not editable by wide‐type LbCas12a nor AsCas12a nucleases in previous reports [15, 16]. These findings indicate that Mb2Cas12a is capable of effectively editing relaxed ATTV PAM sites, thereby further enhancing its utility in plant genome editing.

### CRISPR/Mb2Cas12a system enables multiplex genome editing in cotton with a single crRNA cassette

To evaluate the multiplex genome editing activity of Mb2Cas12a in cotton plants, eight crRNAs were designed to target five endogenous genes (*GhPGF*, *GhCLA1*, *GhFAD*, *GhTAC1*, *GhMYB25*‐like) (Figure 2F). In a total of 79 tested calli or plantlets, the mutation rates of eight target loci ranged from 1.45% to 86.96%. Compared to the single gene editing described earlier at the *GhPGF*‐crRNA1 target site, the editing efficiency of the crRNA1 target site was 39.7% in the multiplex system. Moreover, our results showed that the mutation efficiency for the middle crRNA units (loci 3–4) was relatively lower, ranging from 1.82% to 17.93%. In contrast, the mutation efficiencies were higher for the two ends of the crRNA unit, with 39.7%, 71.47%, and 58.62% of crRNA1, crRNA7, and crRNA8, respectively (Figure 2G).

These results suggest that the editing efficiency of targets in the multiplex genome editing system using the Mb2Cas12a may be influenced by their crRNA‐specific locations [17]. This observation could be attributed to the suitability of the Pol III promoter during the transcription of tRNA and other small RNAs, while it may not be effective for producing long transcripts [6]. Considering the data obtained from 79 calli/plantlets in this report, it is advisable to conduct further tests on a larger number of samples and include more regenerated plants, in addition to calli, to facilitate more comprehensive analysis.

Taken together, the data suggested that Mb2Cas12a is an efficient and precise genome editing tool in cotton plants, which greatly expands the scope of cotton genome editing with a wide temperature adaptability. These findings highlight the potential of Mb2Cas12a as a valuable tool for enhancing genetic improvement and research in cotton.

## CONCLUSION

In summary, we have successfully developed a CRISPR/Mb2Cas12 genome editing system in cotton. This system significantly enhances the capability and extends the scope of cotton genome editing. Meanwhile, we have established an efficient multiplex genome editing system based on Mb2Cas12a, which provides valuable technical tools for the creation of customized cotton germplasm resources and greatly facilitates fundamental biological research in the field. These advancements hold promise for advancing cotton breeding and genetic studies.

## AUTHOR CONTRIBUTIONS

Shuangxia Jin, Xianlong Zhang, and Yong Zhang designed the project. Fengjiao Hui performed experiments, analyzed data, and wrote the manuscript. Xu Tang was involved in the experiments on vector construction. Bo Li, Muna Alariqi, Yongxue Hu, and Guanying Wang were involved in the experiments on genetic transformation and DNA extraction. Qingying Meng and Zhongping Xu performed whole‐genome sequencing data and genome‐wide PAM analysis. Shuangxia Jin, Xianlong Zhang, and Yong Zhang revised the manuscript. All authors have read the final manuscript and approved it for publication.

## CONFLICT OF INTEREST STATEMENT

The authors declare no conflict of interest.

## ETHICS STATEMENT

No animals or humans were involved in this study.

## Supporting information


**Figure S1:** The application of CRISPR/Mb2Cas12a‐mediated genome editing in cotton.
**Figure S2:** Analysis of genetic stability and potential off‐target effects of the Mb2Cas12a in cotton plants.
**Figure S3:** Deep Sequencing result of *GhPGF*‐crRNA1 target site.
**Figure S4:** Deep Sequencing result of *GhPGF*‐crRNA2 target site.
**Figure S5:** Deep Sequencing result of *GhCLA1*‐crRNA1 target site.
**Figure S6:** Deep Sequencing result of *GhCLA1*‐crRNA2 target site.
**Figure S7:** Frequency of deletion position and size induced by Mb2Cas12a nuclease.
**Figure S8:** Editing patterns of different strategies under varying temperature treatments.
**Figure S9:** Analysis of editing efficiency under different temperatures with three different targets.

**Table S1:** The editing efficiency of edited plants.
**Table S2:** Primers used for vector construction.
**Table S3:** Primers used for positive test.
**Table S4:** The crRNA sequences, Barcode primer sequences and amplicon sequences for *GhPGF* gene.
**Table S5:** The crRNA sequences, Barcode primer sequences and amplicon sequences for *GhCLA1* gene.
**Table S6:** The crRNA sequences, primer sequences and amplicon sequences for eight genes.
**Table S7:** Primers used for T0 off‐target sites with Sanger sequencing at *GhPGF*‐crRNA1 target site.
**Table S8:** Primers used for Sanger sequencing at *GhPGF*‐crRNA1 target site.
**Table S9:** Summary of genome‐wide potential off‐targets predictions by Cas‐OFFinder tools for target *GhPGF*‐crRNA1.

## Data Availability

All the sequencing data have been deposited in NCBI under submission number SUB14445092 and SUB14154255, BioProject accession number PRJNA1067522 (https://www.ncbi.nlm.nih.gov/bioproject/PRJNA1067522) and PRJNA1111234 (https://www.ncbi.nlm.nih.gov/bioproject/PRJNA1111234). The data and scripts used have been saved in GitHub https://github.com/fengjiaohui/Mb2Cas12a. Supplementary materials (methods, figures, tables, graphical abstract, slides, videos, Chinese translated version) may be found in the online DOI or iMeta Science http://www.imeta.science/.
