# Supplementary material for: Robust CRISPR/Mb2Cas12a genome editing tools in cotton plants
Source: Imeta. 2024 Jun 4;3(3):e209. doi: 10.1002/imt2.209 (PMC11183160; doi:10.1002/imt2.209)
Supplement: Supplementary file 1 — Figure S1: The application of CRISPR/Mb2Cas12a‐mediated genome editing in cotton. Figure S2: Analysis of genetic stability and potential off‐target effects of the Mb2Cas12a in cotton plants. Figure S3: Deep Sequencing result of GhPGF‐crRNA1 target site. Figure S4: Deep Sequencing result of GhPGF‐crRNA2 target site. Figure S5: Deep Sequencing result of GhCLA1‐crRNA1 target site. Figure S6: Deep Sequencing result of GhCLA1‐crRNA2 target site. Figure S7: Frequency of deletion position and size induced by Mb2Cas12a nuclease. Figure S8: Editing patterns of different strategies under varying temperature treatments. Figure S9: Analysis of editing efficiency under different temperatures with three different targets. [file IMT2-3-e209-s002.docx]

**Supplementing information to**

**Robust CRISPR/Mb2Cas12a genome editing tools in cotton plants**

**Running title**: A novel cotton genome editing system

^1^Hubei Hongshan Laboratory, National Key Laboratory of Crop Genetic Improvement, Huazhong Agricultural University, Wuhan, 430070, China

^2^Chongqing Key Laboratory of Plant Resource Conservation and Germplasm Innovation, Integrative Science Center of Germplasm Creation in Western China (Chongqing) Science City, School of Life Sciences, Southwest University, Chongqing, 400715, China

^3^Institute of Nuclear and Biological Technology, Xinjiang Academy of Agricultural Sciences, Xinjiang Key Laboratory of Crop Biotechnology, Urumqi, 830091, China

*Correspondence: [jsx@mail.hzau.edu.cn](mailto:jsx@mail.hzau.edu.cn) (Shuangxia Jin), [xlzhang@mail.hzau.edu.cn](mailto:xlzhang@mail.hzau.edu.cn) (Xianlong Zhang) and <zhangyong916@swu.edu.cn> (Yong Zhang)

**Supplementary materials and methods**

**Plant material and growth condition**

The cotton (*G. hirsutum*) plants used in this study were upland cotton Jin668 [1], and after sterilization, the cotton seeds were placed on a sterile seedling culture medium at 28 ℃ in the dark for 5 to 7 days for cotton stable transformation. The obtained editing plants are planted in the greenhouse.

**Construction of the vectors**

All vectors used in this study were constructed using Gibson assembly or Golden Gate assembly. The codon-optimized Mb2Cas12a protein was synthesized and assembled with the OsUbi2 promoter and Nos terminator using Golden Gate assembly, resulting in the Mb2Cas12a entry vector pTX1365. PCR fragments containing GhU6.7 promoter-tRNA and crRNA scaffold-lacZ-tRNA-TTTTTTT or the OsUbi2 promoter-Hammerhead ribozyme, crRNA scaffold, and lacZ-HDV ribozyme-Nos terminator were amplified and then inserted into AarI-linearized pMOD_B0000a vector using Gibson assembly, yielding the crRNA entry vectors *pGhU6.7*-crRNA (pTX1363) and *pOsUbi2*-crRNA (pTX1364). To generate the final Mb2Cas12a backbone, the Mb2Cas12a entry vectors and crRNA entry vectors, and pMOD_C0000a were assembled into the T-DNA backbone pTRANS_220d (Addgene Plasmid # 91114) to generate cotton Mb2Cas12a backbones for both Strategy 1 and 2 using Golden Gate assembly. To construct a cotton STU Mb2Cas12a backbone, the OsUbi2 promoter and Mb2Cas12a were amplified and assembled into pSTU_A vector using Gibson assembly, generating pSTU_A-Mb2Cas12a. Similarly, the polyA-Hammerhead ribozyme, crRNA scaffold, and lacZ-HDV ribozyme-Nos terminator were amplified and then inserted into BsaI-linearized pSTU_B vector using Gibson assembly, generating pSTU_B-crRNA. To generate cotton Mb2Cas12a backbones for Strategy 3 using Golden Gate assembly, pSTU_A-Mb2Cas12a, pSTU_B-crRNA, and pMOD_C0000a were assembled into the T-DNA backbone pTRANS_220d (Addgene Plasmid # 91114). Finally, the final T-DNA vectors were prepared by inserting annealed oligos or PCR fragments into the cotton Mb2Cas12a backbones using Golden Gate assembly (Table S2).

***Agrobacterium*-mediated cotton transformation**

All vectors were transformed into *Agrobacterium* strain GV3101 (kanamycin as a selectable marker) via electroporation. The cotton hypocotyls were cut into 0.5−1 cm long, transferred to the activated agrobacterium bacterial solution, stood for 3−5 min, dispersed in the co-culture medium with filter paper, darkened for 36−48 hours, then transferred to 50 mg/L kana screening medium. Change the medium every 3−4 week. After 3−4 months, the resistant callus was selected into the IBA medium and finally transferred to the 1/2 MS medium to obtain full transgenic plants. The detailed protocol for cotton genetic transformation was described in our previous publications [1, 2−5].

**Mutation analysis of on-target sites by Sanger sequencing**

Genomic DNA was extracted from young leaves of T0 and T1 transgenic cotton plants using the CTAB method [6]. Specific primers for Mb2Cas12a and crRNA sequences were used to check and confirm transgenics (Table S3). The targeted sites were amplified using site-specific primers, and the amplicons were ligated in pGEMT-Easy vector using T4 DNA ligase (Promega, Madison, USA). The plasmid containing the amplicons was transformed into E. coli by heat shock. Positive clones were used for DNA Sanger sequencing and quantified using BioEdit 7.2 software (<https://bioedit.software.informer.com/7.2/>).

**On-target mutation detection in transgenic cotton by next-generation sequencing**

Barcode-based Next Generation Sequencing has been used for genotyping of target genes in animals and plants [7]. This method was used to detect target editing efficiency. To track all sequencing data back to a single original transgenic plant/sample, a pair of 6 base combinations was designed as the barcode tag for each sample [8], using our own Python script. Each pair of labels was added to the 5’ ends of the forward and reverse primers to allow amplification of the target sequence (Tables S4−S6). The corresponding barcode primers were used for PCR amplification of independent positive samples, and the resulting PCR products (~200−220bp) were mixed in equal amounts and purified (OMEGA kit, D2500-02). The collected DNA fragments were sequenced by Illumina sequencing and the raw data were filtered to remove low-quality reads and adapters using FastQC [9], and clean reads will be used for further analysis. Demultiplexing was processing where reads from FASTQ sequencing files were assigned to each sample based on the barcode tags. CRISPResso2 was used to analyze the editing efficiency and mutation types [10].

**Detection of off-target mutations by Sanger sequencing and whole genome** **sequencing**

We used the online website Cas-OFFinder (<http://www.rgenome.net/cas-offinder/>) to predict the potential off-target sites, amplified the sequence of interest using site-specific primers, and ligated the amplicon to the pGEMT-Easy vector using T4 DNA ligase (Promega, Madison, USA). Top10 was used to convert the vector to an E. coli strain, and positive clones were sequenced for DNA Sanger sequencing (Tables S7, S8). Besides, Whole genome sequencing (WGS) was performed on Mb2Cas12a edited plant (M237), wild type (WT, Jin668), positive control (PC) plant (containing T-DNA insertion with Mb2Cas12a cassette but without editing) and negative control (NC) plant (following tissue culture and plant generation but without T-DNA insertion). The genome DNA were extracted using the TIANGEN Plant Genomic DNA Kit (TIANGEN, Cat#DP305-03) and was sequenced using the Illumina NovaSeq sequencer (the average depth being 77.5 ×). The potential off-target sites were transformed into Jin668 genome via blastn with parameters ‘-task blastn-short -word_size 7 -evalue 1’. The genome of edited plants was compared with the genomes of WT plants and positive plants to filter out genotypic and somaclonal background variation following our previous report [11]. A total of 438 (7 nt mismatch in DNA length) potential off-target sites were predicted for *GhPGF*-crRNA1 using the Cas-OFFinder (Table S9). The 11 most likely off-target sites (OT1−OT11) were selected for crRNA and compared with the edited plant, the WT, the NC, and the PC plants. We observed obvious fragment deletion at the target site, while there were no deletions at the predicted off-target sites by Integrative Genomics Viewer (IGV) [12], which was consistent with our target Sanger sequencing data (Figure S2).

To further evaluate potential off-target mutations caused by Mb2Cas12a, a caller tool GATK/4.3.0.0 [13] with strict parameters was applied to obtain high concordance variations. For the M237 plant, a total of 88,319 indels and 122,920 SNPs were detected compared with the WT Jin668 reference genome. We also used data from negative control plant and WT plant as a control for the evaluation of somaclonal variation following tissue culture or genetic background variation of explants. The result showed that the Mb2Cas12a-induced mutations (on-target editing) are solely present in edited plant, not in WT and negative control plants. In the PC plant, there were some SNPs (base substitutions) mutations, we suggested that these mutations were derived from somaclonal and/or germline variations during the plant tissue culture. In total, 27,253 unique indels and 14,949 unique SNPs were detected in the M237 plant. After filtering the shared variations between M237 and PC, the remaining individual variations (4,405 indels and 2,087 SNPs) were filtered for further detection of Mb2Cas12a-induced off-target mutations, only account for 5% indels and 1.7% SNPs of the total variation (Figure S2). We searched these 438 sites on the Jin668 reference genome by “blastn” that retrieved 2182 position information, then these positions were mapped into with total indels and SNPs to see whether they are overlapped or not.

### Bioinformatics analysis for variant calling

The reference allotetraploid cotton genome (*Gossypium hirsutum*, Jin668) and its annotation were downloaded from CottonGen (<https://www.cottongen.org/>). First, the raw reads were filtered with Trimmomatic (Version 0.39, MINLEN:50). Then, clean reads were mapped Jin668 genome using BWA-MEM (v0.7.17). Only those reads with high mapping quality against the reference genome (-F 4 -q 30) were kept. HaplotypeCaller from GATK was used to generate a genomic variant call format file (gVCF) for each sample, which was used for joint genotyping among all samples (via “GenotypeGVCFs” from GATK). The SNPs and indels were filtered with parameter (QD < 2.0 || MQ < 40.0 || FS > 60.0 || SOR > 3.0 || MQRankSum < -12.5 || ReadPosRankSum < -8.0).

**Temperature treatment for cotton plants**

We exposed the transgenic T0 and T1 plants to temperatures of 22 ℃, 25 ℃, 28 ℃, and 32 ℃ for 7 days. We then selected the tenderest leaf at the top to determine editing efficiency. DNA was extracted from young leaves of plants using the CTAB method. The collected DNA fragments were sequenced by Illumina sequencing. The analysis method of deep sequencing data was as described above.

**Genome-wide PAM analysis**

The cotton cultivar Jin668 reference genome sequence was obtained from <http://cotton.hzau.edu.cn/EN/Download.htm>. Using the 'locate' subcommand of the SeqKit software to perform PAM statistics, including both the NGG, TTV, and TTTV sequence and its reverse complementary sequence [14].

**Statistical analysis**

The data were analysed with the GraphPad Prism 9.0 software. Statistical differences were analyzed using Student's t-test. The threshold for significant was set to *p*-value  <  0.05. The figures were made using Office Excel and Adobe Illustrator software.

**Supplementary figure and table legends**

Figure S1 The application of CRISPR/Mb2Cas12a-mediated genome editing in cotton.

Figure S2 Analysis of genetic stability and potential off-target effects of the Mb2Cas12a in cotton plants.

Figure S3 Deep Sequencing result of *GhPGF*-crRNA1 target site.

Figure S4 Deep Sequencing result of *GhPGF*-crRNA2 target site.

Figure S5 Deep Sequencing result of *GhCLA1*-crRNA1 target site.

Figure S6 Deep Sequencing result of *GhCLA1*-crRNA2 target site.

Figure S7 Frequency of deletion position and size induced by Mb2Cas12a nuclease.

Figure S8 Editing patterns of different strategies under varying temperature treatments.

Figure S9 Analysis of editing efficiency under different temperatures with three different targets.

Table S1 The editing efficiency of edited plants.

Table S2 Primers used for vector construction.

Table S3 Primers used for positive test.

Table S4 The crRNA sequences, Barcode primer sequences and amplicon sequences for *GhPGF* gene.

Table S5 The crRNA sequences, Barcode primer sequences and amplicon sequences for *GhCLA1* gene.

Table S6 The crRNA sequences, primer sequences and amplicon sequences for eight genes.

Table S7 Primers used for T0 off-target sites with Sanger sequencing at *GhPGF*-crRNA1 target site.

Table S8 Primers used for Sanger sequencing at *GhPGF*-crRNA1 target site.

Table S9 Summary of genome-wide potential off-targets predictions by Cas-OFFinder tools for target *GhPGF*-crRNA1.

**
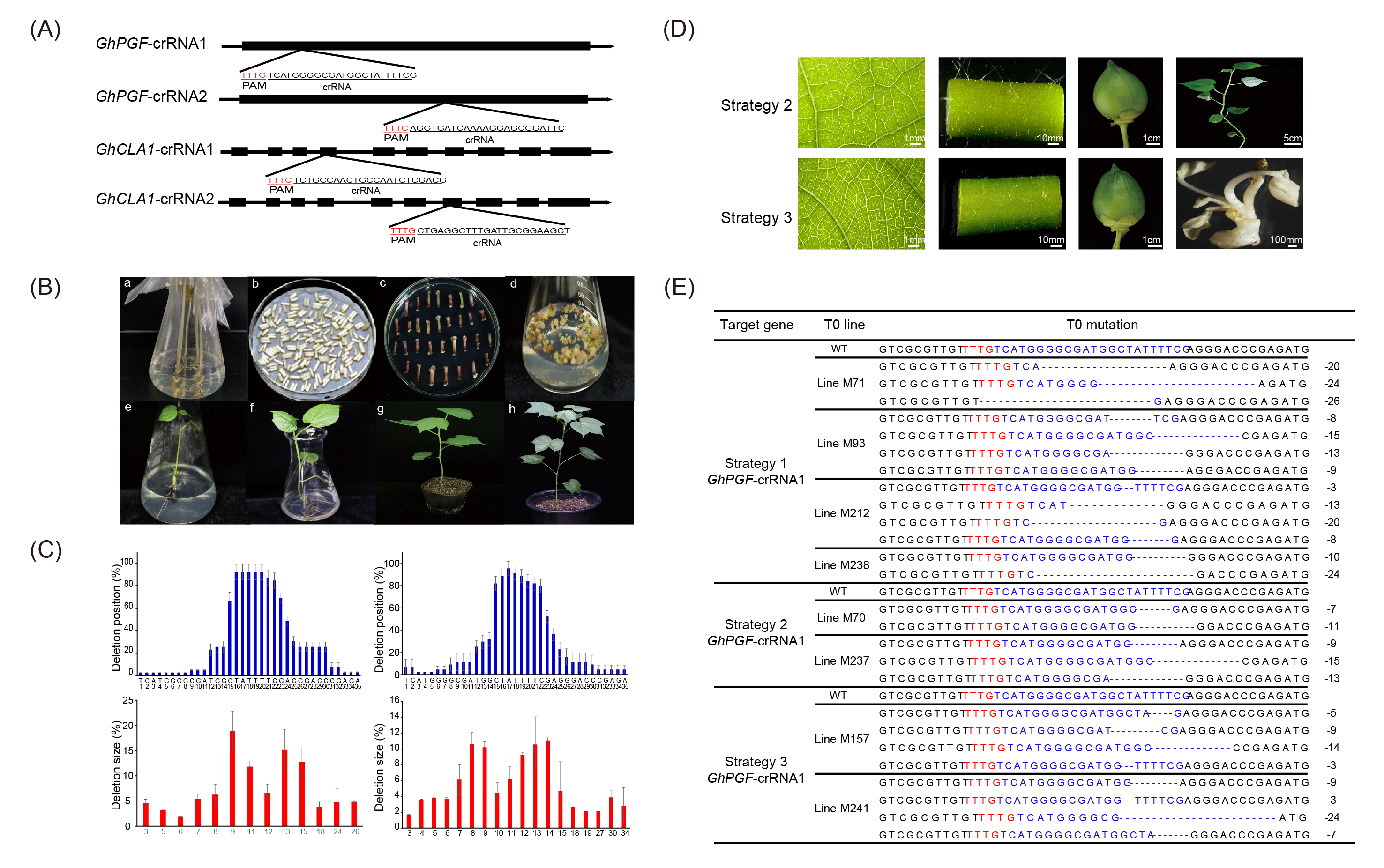
**

**Figure S1 The application of CRISPR/Mb2Cas12a-mediated genome editing in cotton**. (A) The target site of crRNAs in the coding region of *GhPGF* and *GhCLA1* and all the target DNA sequences were listed. Red, protospacer adjacent motif (PAM) sequences. Black, the target DNA sequences. (B) Transgenic cotton plants were obtained through *Agrobacterium*-mediated transformation and cotton tissue culture and somatic embryogenesis. (C) Frequency of deletion position and size of DNA deletion at the target site of *GhPGF*-crRNA1 induced by Mb2Cas12a with Strategy 2 and Strategy 3. (D) Phenotype of the T0 cotton plants with the target mutations in *GhPGF* and *GhCLA1* with Strategy 2 and Strategy 3. The black dots in each picture are the gossypol glands of cotton. The leaves of T0 knockout plants with *GhCLA1*-crRNA2 target site were bleached. (E) Targeted editing profiles of T0 plants at the *GhPGF*-crRNA1 site using Strategy 1 to 3 by Sanger sequencing.

**
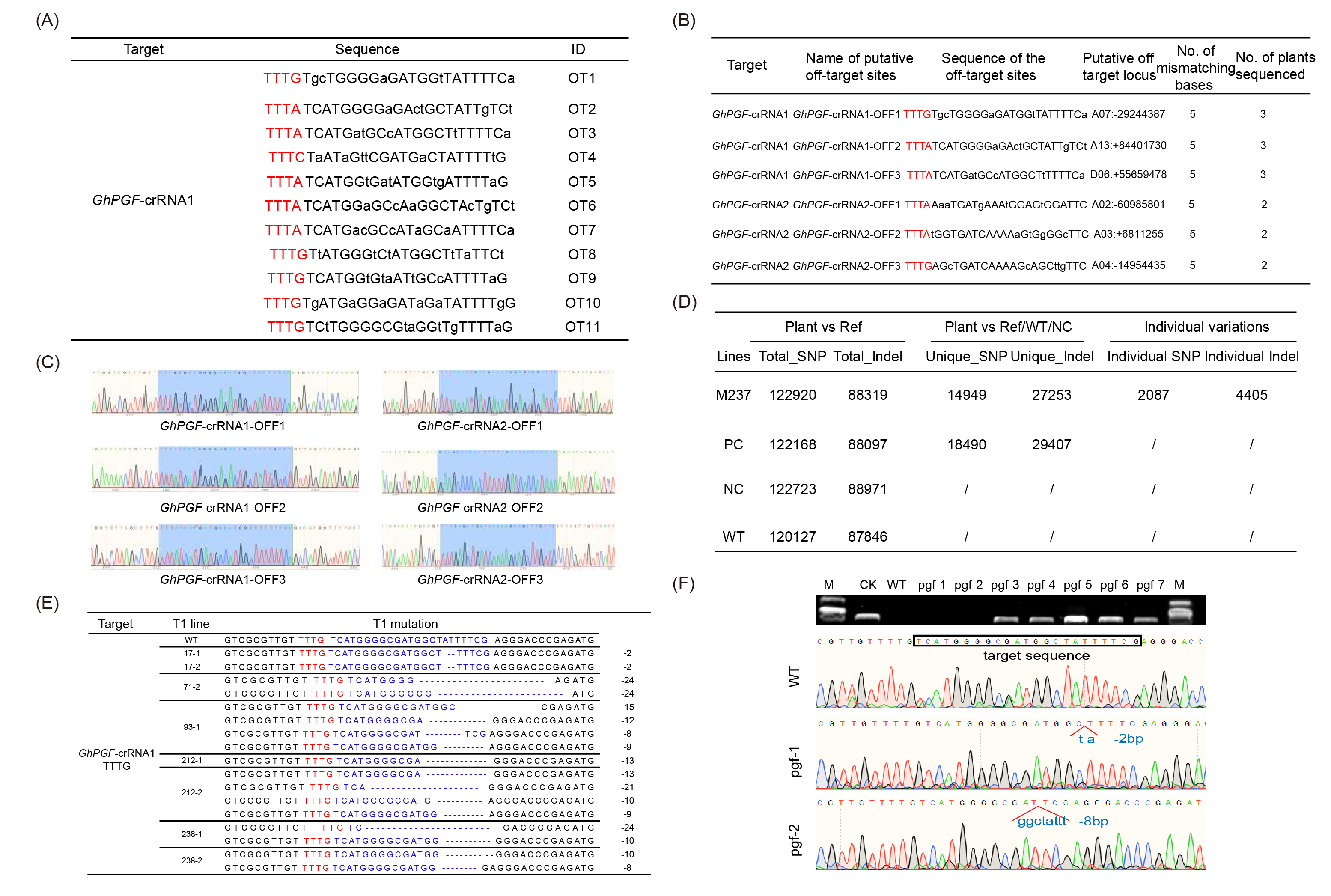
**

**Figure S2 Analysis of genetic stability and potential off-target effects of the Mb2Cas12a in cotton plants**. (A) The eleven potential off-target sites were predicted by the Cas-OFFinder website with *GhPGF-*crRNA1. PAM motif is shown in red; mismatching bases are shown in lowercase letters. (B) Three potential off-target sites were predicted at *GhPGF-*crRNA1 and *GhPGF*-crRNA2 target sites by the Cas-OFFinder website, respectively. PAM motif is shown in red; mismatching bases are shown in lowercase letters. (C) Detection of Off-target effects at six predicted potential off-target sites in five independent T0 plants by Sanger sequencing. (D) Indels and SNPs of Mb2Cas12a edited cotton plant M237, wild type (WT, Jin668), positive control (PC) plant, negative control (NC) plant revealed by whole-genome sequencing. The ‘Plant vs Ref’ represents the high confidence variations of the edited plant compared with Jin668 reference genome. The ‘Plant vs Ref/WT/NC’ represents the variations of transgenic-edited plant compared with Jin668, WT, and NC including edited plant and PC. Individual variation indicated the specific variations of the edited plant. Blastn:-task blastn-short -word_size 7 -evalue 1. (E) Target mutation profiles of T1 lines at the *GhPGF*-crRNA1 site. Genotype analysis revealed faithful inheritance of the mutations from T0 plants in T1 lines. Only DNA deletions were detected of T1 plants. (F) Identification of transgene-free and glandless T1 cotton plants. In the upper panel, the presence of Mb2Cas12a sequences was observed in five plants, but not in pgf-1 and pgf-2 plants. (M) DL2000 Marker; WT: Wild type; CK: Plasmid control. Lower panel: The edited *GhPGF* sequences in the two mutated plants (pgf-1 and pgf-2) showed a 2 bp indel (-ta-) and an 8 bp indel (-ggctattt-).


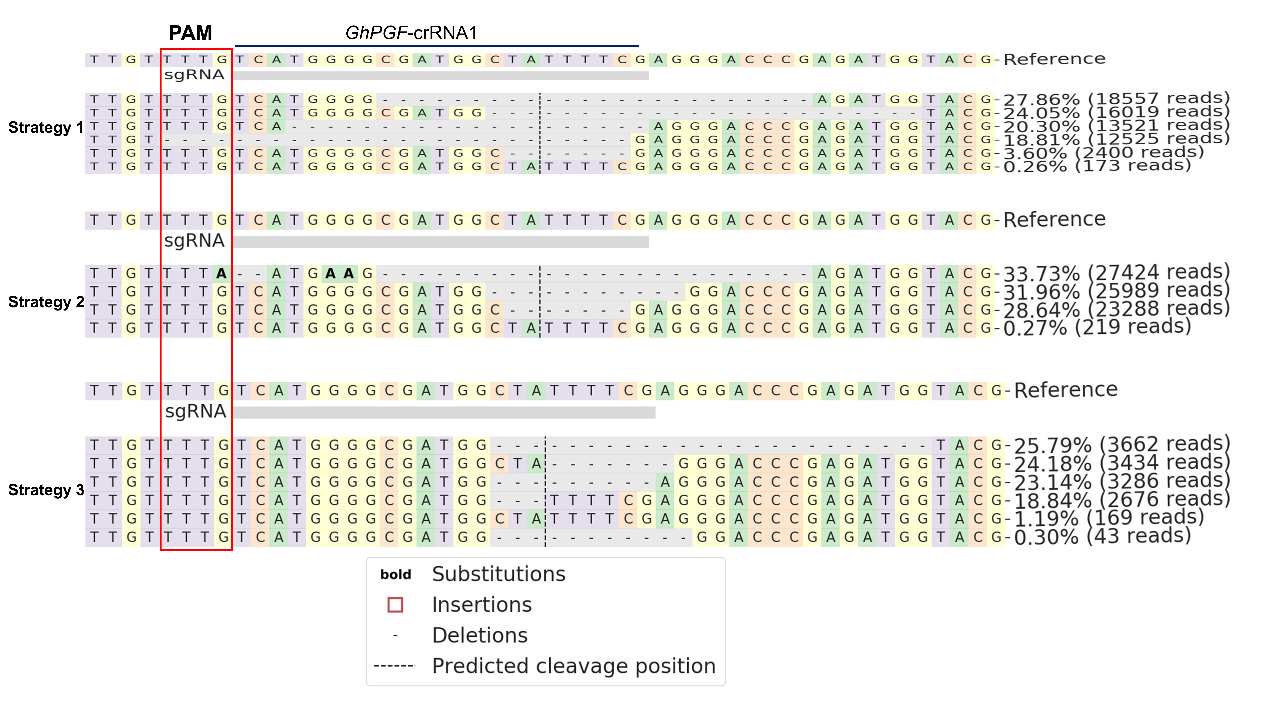


**Figure S3 Deep Sequencing result of *GhPGF*-crRNA1 target site**.


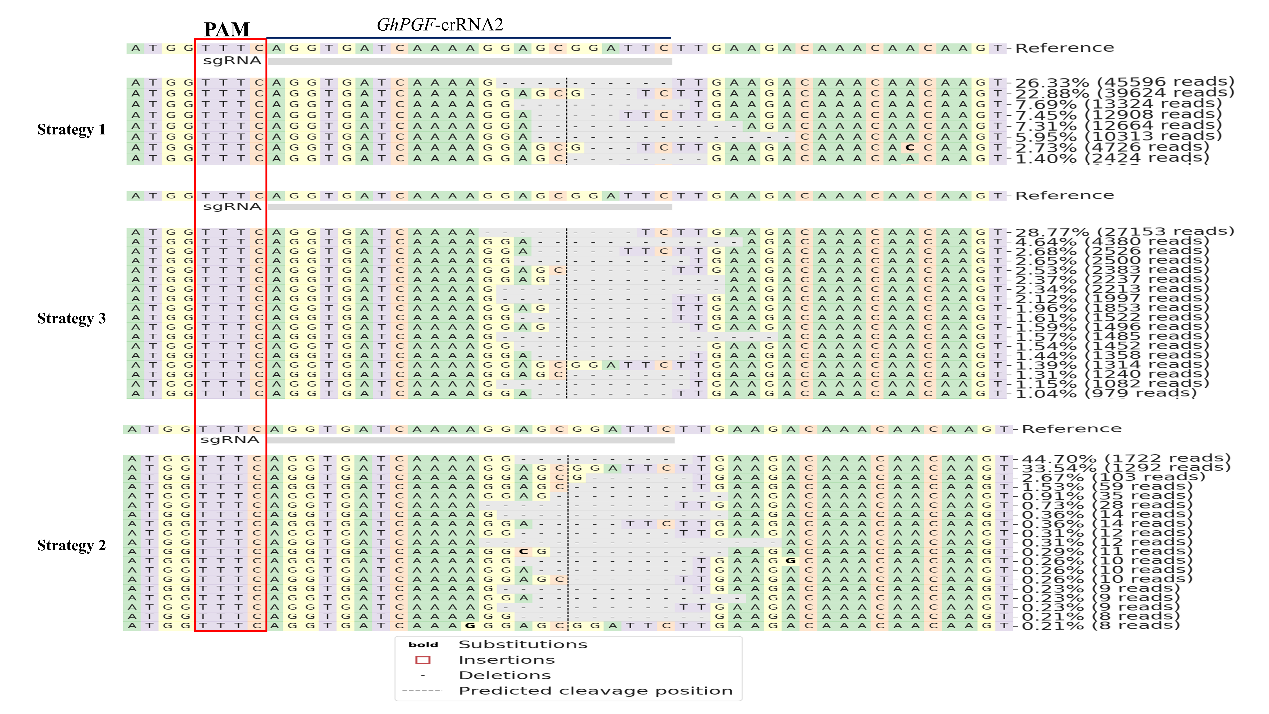


**Figure S4 Deep Sequencing result of *GhPGF*-crRNA2 target site**.


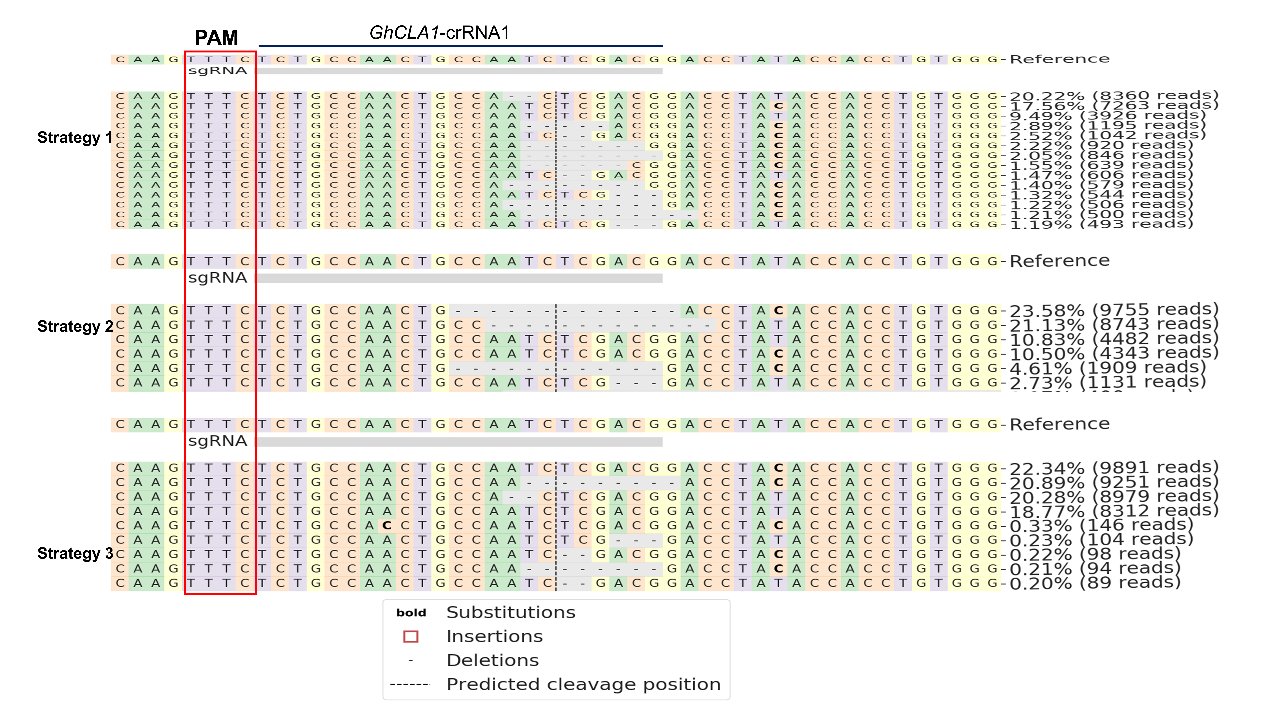


**Figure S5 Deep Sequencing result of *GhCLA1*-crRNA1 target site**.


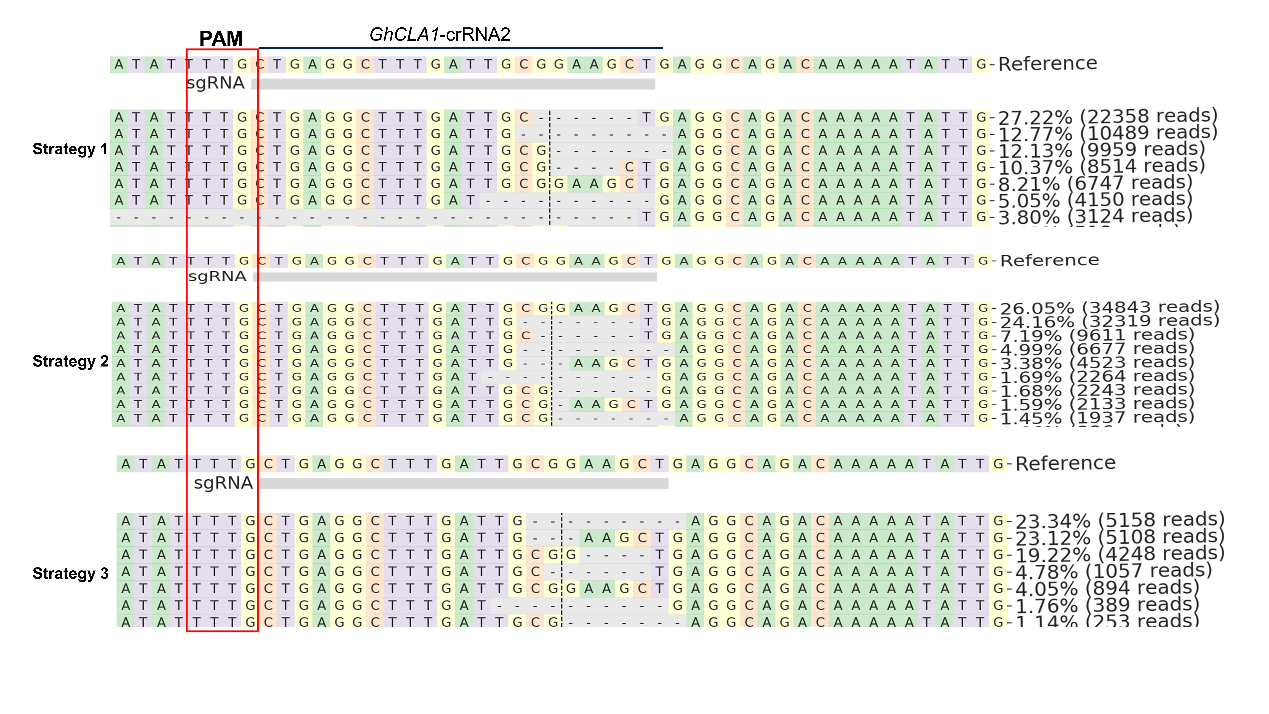


**Figure S6 Deep Sequencing result of *GhCLA1*-crRNA2 target site**.

**
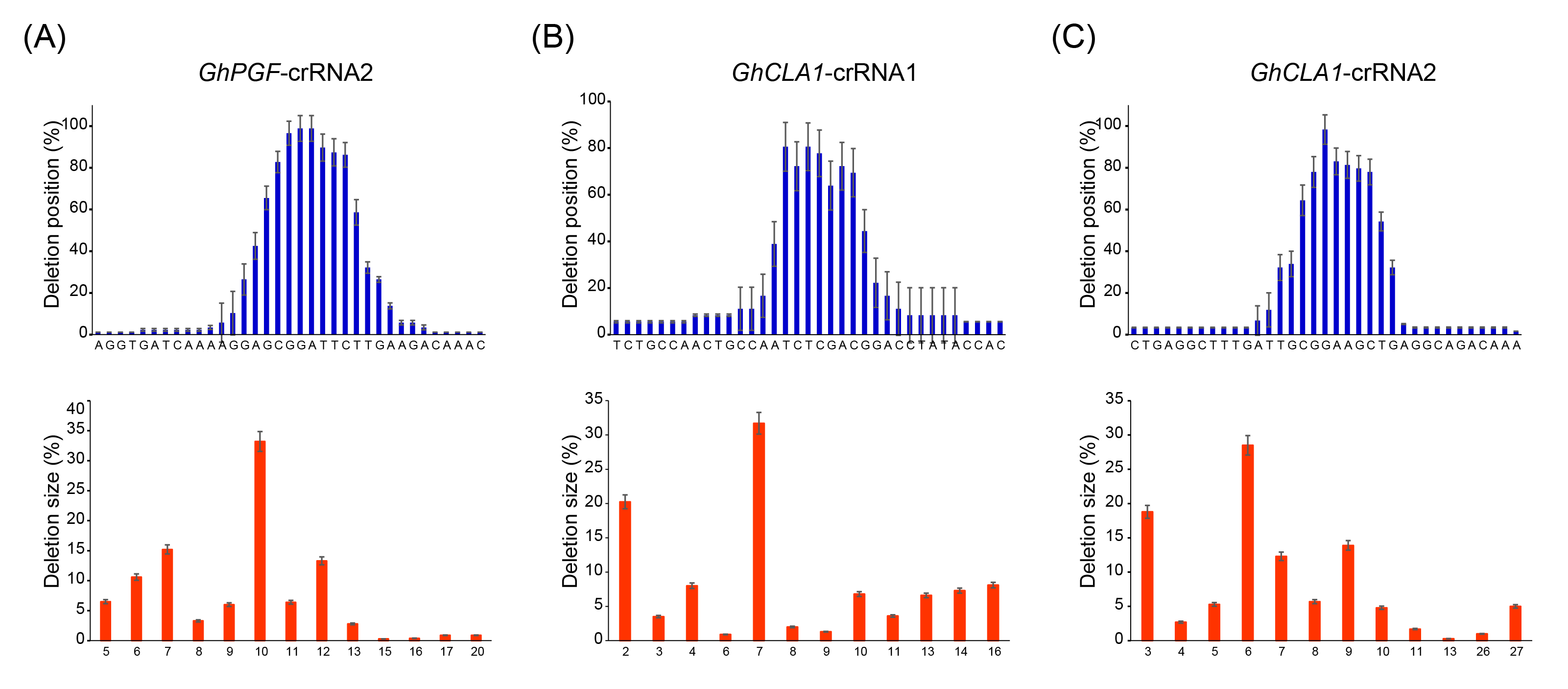
**

**Figure S7 Frequency of deletion position and size induced by Mb2Cas12a nuclease**. (A) Frequency of DNA deletion position and size at the target site *GhPGF*-crRNA2. (B) Frequency of DNA deletion position and size at the target site *GhCLA1*-crRNA1. (C) Frequency of DNA deletion position and size at the target site *GhCLA1*-crRNA2.

**
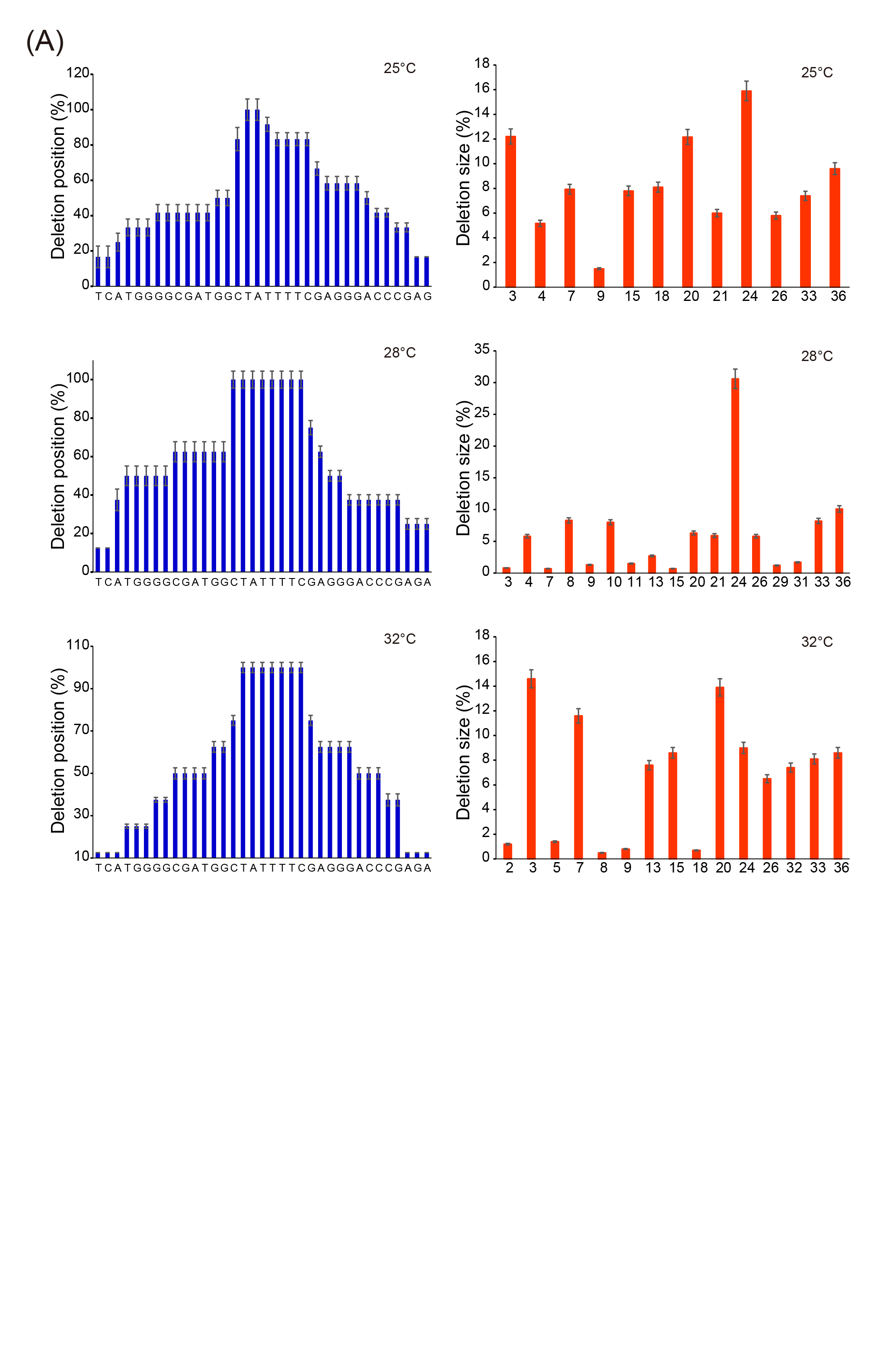

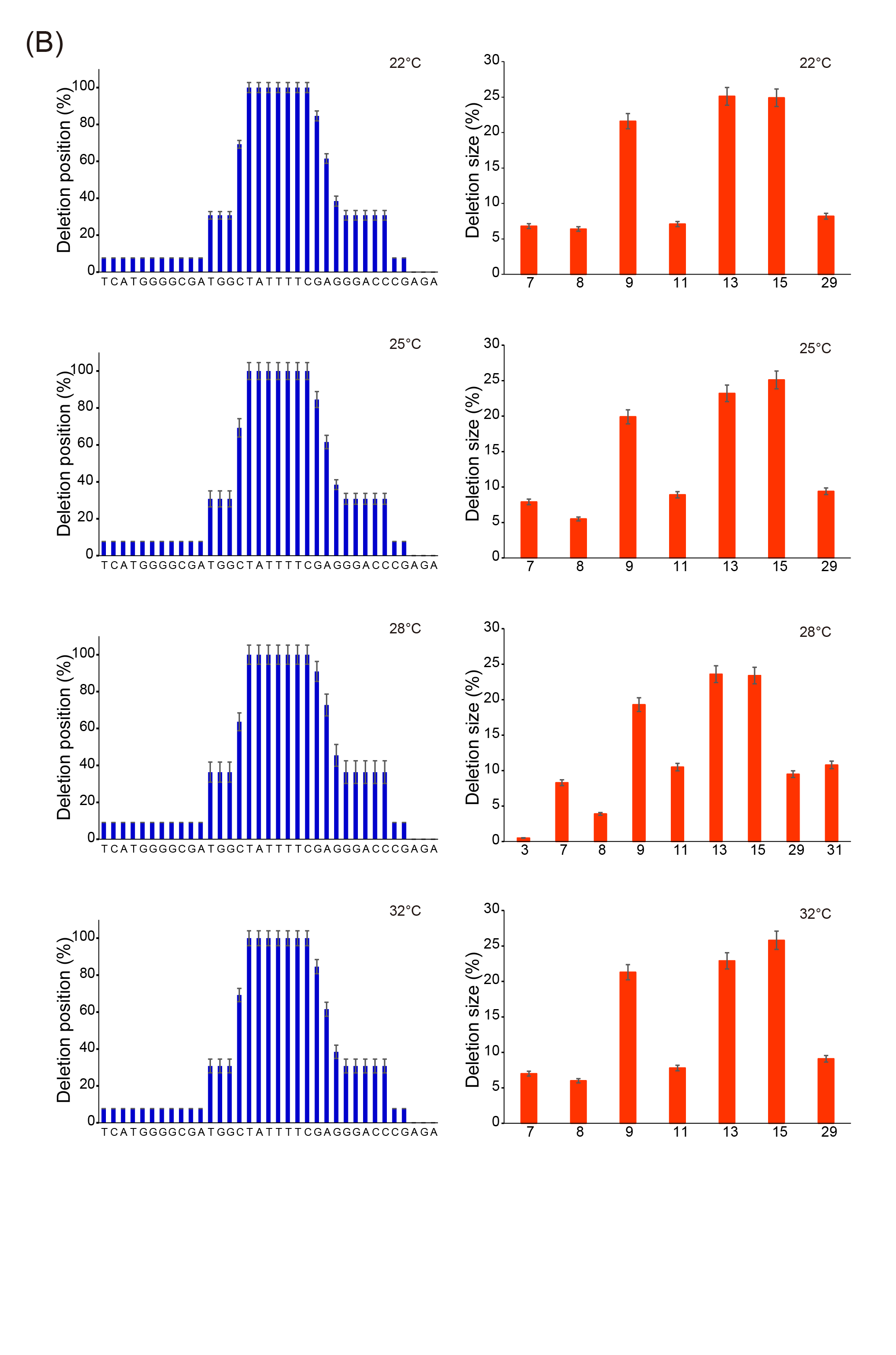

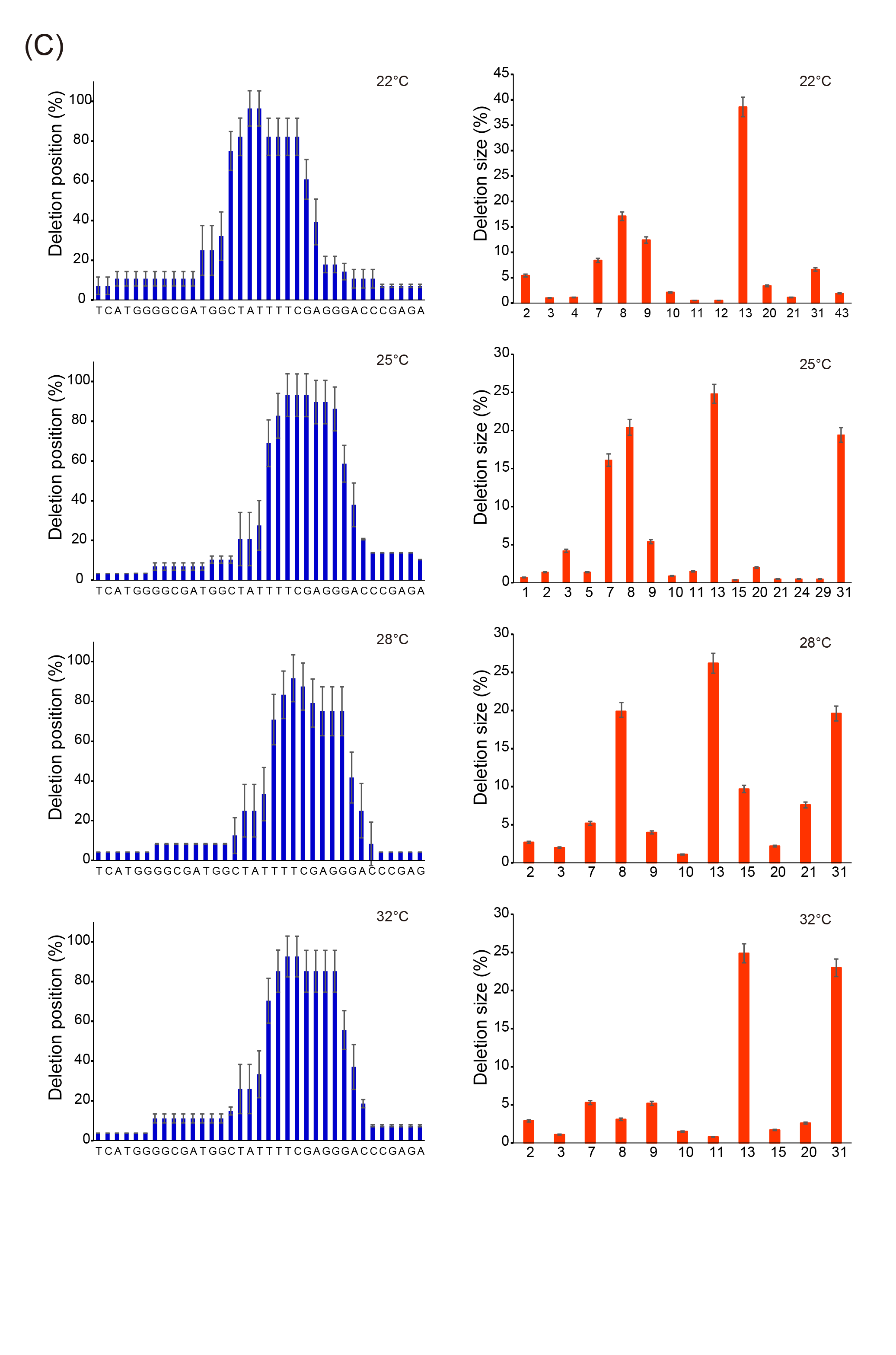
**

**Figure S****8 Editing patterns of different strategies under varying temperature treatments.** (A) Temperature treatments of *GhPGF*-crRNA1 with Strategy 1. (B) Temperature treatments of *GhPGF*-crRNA1 with Strategy 2. (C) Temperature treatments of *GhPGF*-crRNA1 with Strategy 3.

**
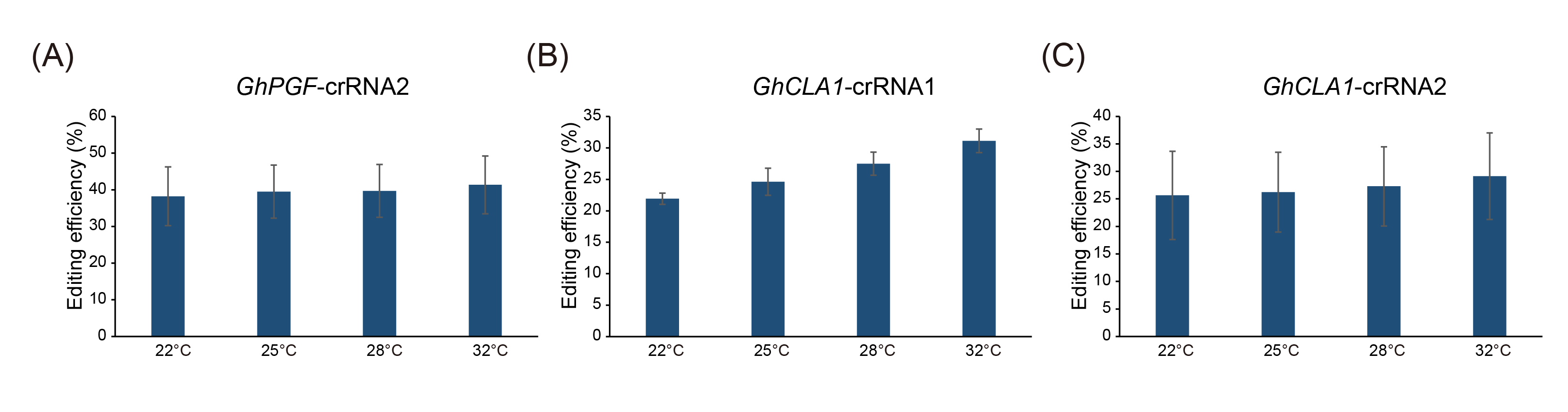
**

**Figure S9 Analysis of editing efficiency under different temperatures with three different targets**. (A) Temperature treatments of *GhPGF*-crRNA2 plants. (B) Temperature treatments of *GhCLA1*-crRNA1 plants. (C) Temperature treatments of *GhCLA1*-crRNA2 plants. Values are means ± Standard Deviation (n = 3).

**REFERENCES**

1. Li, Jianying, Maojun Wang, Yajun Li, Qinghua Zhang, Keith Lindsey, Henry Daniell, Shuangxia Jin, et al. 2019. “Multi-omics analyses reveal epigenomics basis for cotton somatic embryogenesis through successive regeneration acclimation process.” *Plant Biotechnology Journal* 17:435-450. <https://doi.org/10.1111/pbi.12988>

2. Zhu, Xiangqian, Zhongping Xu, Guanying Wang, Yulong Cong, Lu Yu, Ruoyu Jia, Yuan Qin, et al. 2023. “Single-cell resolution analysis reveals the preparation for reprogramming the fate of stem cell niche in cotton lateral meristem.” *Genome Biology* 24:194. <https://doi.org/10.1186/s13059-023-03032-6>

3. Sun, Lin, Muna Alariqi, Yaxin Wang, Qiongqiong Wang, Zhongping Xu, Muhammad Naeem Zafar, Guangqin Yang, et al. 2023. “Construction of host plant insect-resistance mutant library by high-throughput CRISPR/Cas9 system and identification of a broad-spectrum insect resistance gene.” *Advanced Science* 11(4):2306157. <https://doi.org/10.1002/advs.202306157>

4. Liang, Sijia, Jing Luo, Muna Alariqi, Zhongping Xu, Aoli Wang, Muhammad Naeem Zafar, Jun Ren, et al. 2021. “Silencing of a LIM gene in cotton exhibits enhanced resistance against Apolygus lucorum.” *Journal of Cellular Physiology* 236:5921-5936. <https://doi.org/10.1002/jcp.30281>

5. Sun, Lin, Muna Alariqi, Yi Zhu, Jianying Li, Zelin Li, Qing Wang, Yajun Li, et al. 2018. “Red fluorescent protein (DsRed2), an ideal reporter for cotton genetic transformation and molecular breeding.” *The Crop Journal* 6:366-376. <https://doi.org/10.1016/j.cj.2018.05.002>

6. Porebski, Sue, L. Grant Bailey, Bernard R. Baum. 1997. “Modification of a CTAB DNA extraction protocol for plants containing high polysaccharide and polyphenol components.” *Plant Molecular Biology Reporter* 15:8-15. <https://doi.org/10.1007/BF02772108>

7. Loman, Nicholas J., Raju V. Misra, Timothy J. Dallman, Chrystala Constantinidou, Saheer E. Gharbia, John Wain, Mark J. Pallen. 2012. “Performance comparison of benchtop high-throughput sequencing platforms.” *Nature Biotechnology* 30:434-439. <https://doi.org/10.1038/nbt.2198>

8. Ståhlberg, Anders, Paul M. Krzyzanowski, Matthew Egyud, Stefan Filges, Lincoln Stein, Tony E. Godfrey. 2017. “Simple multiplexed PCR-based barcoding of DNA for ultrasensitive mutation detection by next-generation sequencing.” *Nature Protocols* 12:664-682. <https://doi.org/10.1038/nprot.2017.006>

9. Andrews, S. 2014. “FastQC A Quality Control tool for High Throughput Sequence Data.” [*http://www.bioinformatics.babraham.ac.uk/projects/fastqc/*](http://www.bioinformatics.babraham.ac.uk/projects/fastqc/)

10. Clement, Kendell, Holly Rees, Matthew C. Canver, Jason M. Gehrke, Rick Farouni, Jonathan Y. Hsu, Mitchel A. Cole, et al. 2019. “CRISPResso2 provides accurate and rapid genome editing sequence analysis.” *Nature Biotechnology* 37:224-226. <https://doi.org/10.1038/s41587-019-0032-3>

11. Li, Jianying, Hakim Manghwar, Lin Sun, Pengcheng Wang, Guanying Wang, Hanyan Sheng, Jie Zhang, et al. 2019. “Whole genome sequencing reveals rare off-target mutations and considerable inherent genetic or/and somaclonal variations in CRISPR/Cas9-edited cotton plants.” *Plant Biotechnology Journal* 17:858-868. <https://doi.org/10.1111/pbi.13020>

12. Robinson, James T., Helga Thorvaldsdóttir, Wendy Winckler, Mitchell Guttman, Eric S. Lander, Gad Getz, Jill P. Mesirov. 2011. “Integrative genomics viewer.” *Nature Biotechnology* 29:24-26. <https://doi.org/10.1038/nbt.1754>

13. McKenna, Aaron, Matthew Hanna, Eric Banks, Andrey Sivachenko, Kristian Cibulskis, Andrew Kernytsky, Kiran Garimella, et al. 2010. “The Genome Analysis Toolkit: A MapReduce framework for analyzing next-generation DNA sequencing data.” *Genome Research* 20:1297-1303. <https://doi.org/10.1101/gr.107524.110>

14. Shen, Wei, Shuai Le, Yan Li, Fuquan Hu. 2016. “SeqKit: A Cross-Platform and Ultrafast Toolkit for FASTA/Q File Manipulation.” *PLOS ONE* 11:e0163962. <https://doi.org/10.1371/journal.pone.0163962>
